# Seed to seed variation of proteins of the yellow pea (*Pisum sativum* L.)

**DOI:** 10.1371/journal.pone.0271887

**Published:** 2022-08-04

**Authors:** Mostafa Taghvaei, Rohollah Sadeghi, Brennan Smith

**Affiliations:** 1 Department of Animal, Veterinary, and Food Science, University of Idaho, Moscow, Idaho, United States of America; 2 USDA-ARS-SRRC Food Processing and Sensory Quality, New Orleans, LA, United States of America; Cairo University, EGYPT

## Abstract

The existing variation among pea protein isolates’ functionality limits their application in food formulations. The source and extent of variations among yellow pea protein profiles was assessed in 10 single seeds of two varieties with different size and weight. A new approach was developed to analyze proteins of yellow pea combining three analytical methods of size exclusion chromatography (SEC), reverse phase high performance liquid chromatography (RP-HPLC), and microfluidic SDS-PAGE, to achieve the highest separation resolution. A high variation of protein concentration was observed not only between varieties, but also among seeds of the same variety. Vicilin to legumin ratio was between 2.72–4.19, and 1.70–2.22 among the individual seeds of AC Agassiz and CDC Saffron varieties, respectively. V/L ratio was significantly different among the individual seeds for both varieties. The amount of some protein fractions/subunits were correlated with seeds’ size and weight for AC Agassiz, while such correlations were not observed for CDC Saffron.

## 1. Introduction

Pulses, like yellow peas (*Pisum sativum* L.) are a part of different diets around the world and are gaining more popularity in North America due to the consumer’s demand for new sources of plant proteins that are non-GMO and allergy free [1, 2]. Pulse proteins are particularly important for providing a rich source of essential amino acids and bioactive peptides [3] along with functional properties such as water holding, fat binding, foaming and gelation [4]. This wide range of functionalities gives pulse protein isolates a broad area of application in food products. Many pea protein concentrates and isolates are commercially available and have a great potential as a food ingredient [5]. However, issues in consistency in physicochemical properties have hindered the widespread use of these proteins.

The storage proteins of peas are primarily composed of globulins as defined by the Osborn fractionation scheme [6, 7]. The globulin fraction accounts for ~80% of the total protein of the seed [7]. Of this fraction, there are two key groups of proteins. The first, legumin, is analogous to glycinin in soy (*Glycine max*) and is 360 kDa hexamer [8]. Legumin also contains about 63% less cysteine than glycinin and has been reported to be held together by hydrophobic interactions rather than interprotein disulfide linkages [9, 10]. The second major globular protein of peas is vicilin. Vicilin is analogous to β-conglycinin in soy, and both are ~170 kDa trimeric proteins [9, 11]. The relative amount or ratio between vicilin and legumin is known to influence functional traits like gelling, emulsification, and foaming properties [7].

Regarding functionality in food systems, legumin of peas and glycinin of soy are similar in that greater quantities of these fractions relative to the other storage proteins are known to promote gelling properties [7, 9, 12]. Conversely, vicilin has been characterized as advantageous to foaming properties when in greater concentrations [7, 13]. A minor protein fraction known as convicilin is also present, but in lower concentrations than legumin and vicilin and is known to hinder gel formation [14]. Work by Casey [13] postulated that controlling these ratios would allow for specific physicochemical traits that could lead to further utilization. Tzitzikas, Vincken, De Groot, Gruppen, and Visser [7] took this further, by determining the genetic source of variability in order to determine if individual cultivars may be better suited for specific applications.

While pea protein isolates have entered the food market, finding a place in the market has proved difficult due to a high variation existing among pea protein isolates’ functionality [7]. Inconsistency in the functionality of pea protein isolates from lot to lot is challenging for food processors to have a product with consistent characteristics. The objective of this study was to expand on the work completed by Tzitzikas, Vincken, De Groot, Gruppen, and Visser [7] that determine the seed to seed and the genetic variabilities of pea seed globulins. This was accomplished by combining SEC, RP-HPLC, and microfluidic SDS-PAGE for 2D separation of yellow pea proteins to study the extent of variation in protein profiles among individual seeds of the same cultivar grown in the same location and determine any possible correlations between seeds size and weight with the biochemical properties. Because the root cause of functional variability lies with biochemical variability, defining the extent of protein variation among individual seeds could help to determine the source of variations and will provide a platform to obtain protein isolates with more consistent functionality. The results of this study could help to identify the possible sources of the high variability in the functionality of pea protein isolates and take further steps to minimize the extend of these variations.

## 2. Materials and methods

### 2.1 Sample preparation

**Fig 1:**
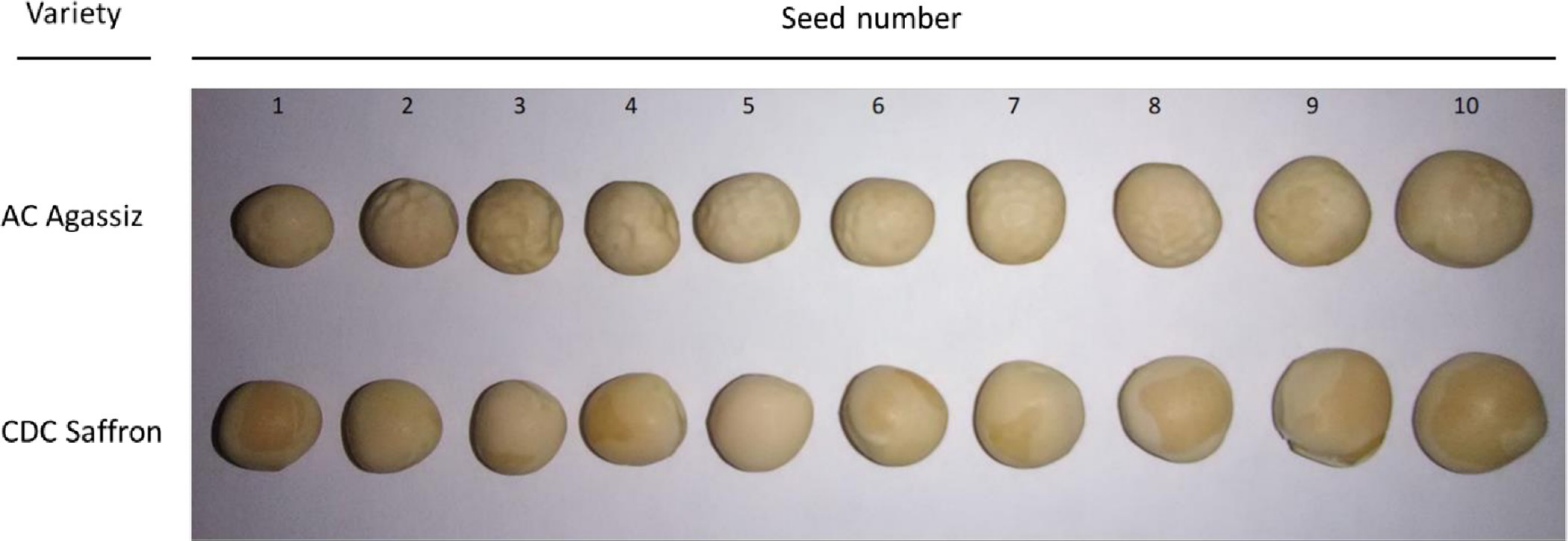
The selected seeds of two varieties, CDC Saffron and AC Agassiz, and their number allocation (1 to 10 from small to large).

For all experiments, two commercially available varieties of dry yellow pea, CDC Saffron and AC Agassiz were used. Seeds were obtained from a seed grower in the 2019 growing season and were grown in the Western regions of Washington, USA. Ten seeds of each variety were selected from the smallest to the largest seed in the batch (Fig 1). After weighing each seed with an analytical balance, their average diameter was determined by three measurements of diameter from top to bottom, side to side, and front to the back, using a digital caliper. Individual seeds were ground (as is) in a mortar and pestle and stored in 2mL centrifuge tubes until analysis.

### 2.2 HPLC system

An Agilent 1100 Series HPLC system equipped with an auto-sampler (model G1313A), and a diode array detector (model G1315B) was used for both RP-HPLC and SEC. Proteins were detected at a wavelength of 214 nm. The OpenLAB CDS (ChemStation Edition) software was used to analyze, integrate, and collect the data.

### 2.3 Size exclusion chromatography

The SEC separations were performed according to the method described by Smith, Bean, Schober, Tilley, Herald, and Aramouni [6] with little modification. Briefly, 30 mg of each ground sample was mixed with 1 mL of 6 M guanidine hydrochloride solution (Fisher Biotech, USA) containing 2% beta mercaptoethanol (BME). The mixture was vortexed for 30 min followed by centrifugation at 15,000 *g*. The supernatant was passed through a 0.45 μm syringe filter (Newway Nylon66, L&Y scientific Inc) and 90 μL was injected into a BioSep S4000 column (5 μm particle size, 7.8 mm ID, and 600 mm long, Phenomenex, USA). The SEC runs were performed using an isocratic flow of 50 mM phosphate-buffered saline (pH = 7) at 1 mL/min. Column temperature was kept at 35°C during the runs.

**Fig 2:**
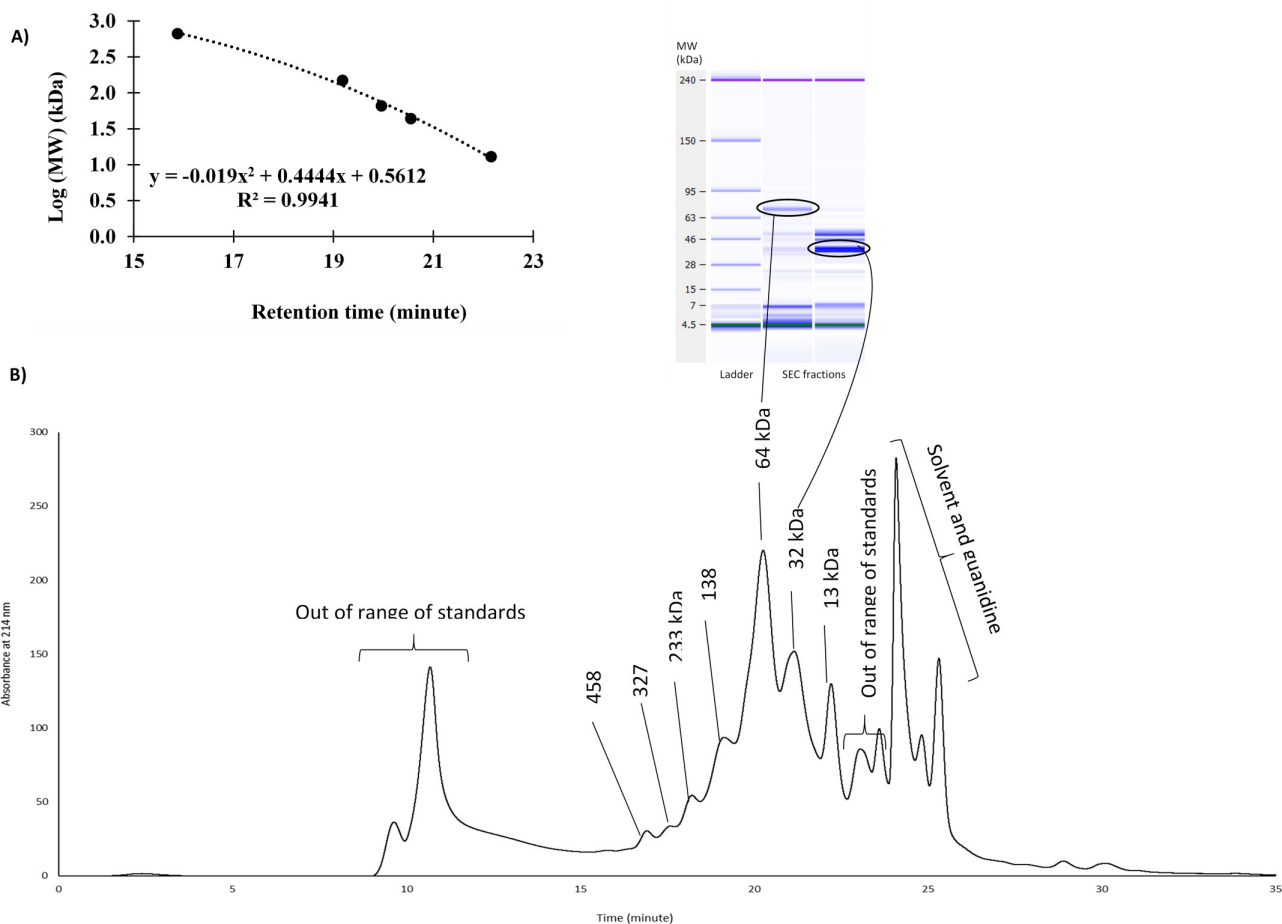
A: The standard curve made with a protein standard mix containing bovine thyroglobulin (670 kDa), Blood γ-globulin (150 kDa), ovalbumin (44 kDa), and Ribonuclease A type I-A from bovine pancreas (13 kDa). B: Size exclusion chromatogram with molecular weight identification of peaks based on the standard curve (Fig 2A) and confirmation of the identifications of 64 and 32 kDa peaks with microfluidic SDS-PAGE.

The estimation of molecular weights was completed using a standard curve made with a protein standard mix (Sigma-Aldrich, USA) containing bovine thyroglobulin (670 kDa), Blood γ-globulin (150 kDa), ovalbumin (44 kDa), and Ribonuclease A type I-A from bovine pancreas (13 kDa) (Fig 2A).

### 2.4 RP-HPLC

The RP-HPLC analysis was performed according to the method described by Taghvaei and Smith [15]. Briefly, 10 mg of each ground sample was mixed with 1 mL of 6 M guanidine hydrochloride solution (Fisher Biotech, USA) containing 2% BME. The mixture was vortexed for 30 min followed by centrifugation at 15,000 *g*. The supernatant was passed through a 0.45 μm syringe filter (Newway Nylon66, L&Y scientific Inc) and 10 μL was injected into the HPLC system described previously.

A Poroshell 300SB-C18 column (5 μm particle size, 2.1 mm ID, and 75 mm long, Agilent Technologies, USA) was used for separation of proteins. The HPLC separation runs were performed using mobile phase A: Water 0.089% TFA (Sigma-Aldrich, USA), and mobile phase B: Acetonitrile 0.089% TFA at the flow rate of 0.7 mL/min. The gradient was from 20% B to 30% B for 10 min, 30% B to 39% B for 20 min, 39% B to 60% B for 10 min, and keeping 60% B for the last 5 min of the run. Column temperature was kept at 55°C.

### 2.5 Two-dimensional separation

Fractions of peaks from SEC were collected using a fraction collector (Agilent 1100 Series) for a duration of 0.3 min for each peak. Each run was repeated 10 times and all 10 fractions of each peak were pooled. To concentrate the amount of proteins in each fraction, 10 kDa molecular weight cutoff centrifugal filters (Millipore, Ireland) were used, except for the last peak of SEC analysis containing proteins smaller than 10 kDa, which centrifugal filters with molecular weight cutoff of 3 kDa were used. The centrifugation was continued until the volume of each concentrated fraction reached 50 μL. Then, 20 μL of each concentrated fraction was injected to the RP-HPLC system according the method described in section 2.4. The coordinates of chromatograms obtained from each fraction were converted into an XYZ format, and a 3D surface plot was generated using OriginPro 8.6 (OriginLab Corporation, USA) software. Four microliters (4 μL) of each concentrated fraction were analyzed by SDS-PAGE microfluidics to confirm molecular weight determinations.

### 2.6 SDS-PAGE microfluidic gel electrophoresis

For microfluidic analysis, 10 mg of each ground sample was mixed with 1 mL of 1% Sodium dodecyl sulfate (SDS) solution (Fisher Biotech, USA) containing 2% BME. The mixture was vortexed for 30 min followed by centrifugation at 15,000 *g* for 5 min. Four microliters (4 μL) of the supernatant were used for microfluidic gel electrophoresis according to the manufacturing company’s assay guide [16]. The 2100 Expert software (Agilent, USA), was used to collect, analyze, and integrate the data. The electropherogram peaks were assigned as described by Tzitzikas, Vincken, De Groot, Gruppen, and Visser [7] to the corresponding protein fragments based on molecular weight (gamma-vicilin (14–16 kDa), alpha-vicilin (19–20 kDa), basic legumin (22 kDa), beta+gamma-vicilin (25–30 kDa), alpha+beta-vicilin (36 kDa), acidic legumin (38–40 kDa), vicilin (48–51 kDa), convicilin (~75 kDa), and lipoxygenase (90–95 kDa)).

### 2.7 Total protein

The total protein content of samples was measured by HPLC according to the method described by Taghvaei and Smith [15] using pea protein isolate as a standard.

### 2.8 Statistical analysis

All experiments were performed in triplicates and the average value (±standard deviation) was reported for each sample/seed. All graphs were made by Microsoft Excel (Microsoft Office 365 ProPlus). Statistical analyses (ANOVA, MANOVA and Pearson correlation) were performed with IBM SPSS 26.0.

## 3. Results and discussion

### 3.1 Size exclusion chromatography

The proteins were initially separated by SEC according to hydrodynamic radii. An estimation of the molecular weights using a standard curve (Fig 2A) allowed for identification of 7 peaks that are shown in Fig 2B. The molecular weight determination of the two peaks with the highest intensities (64 and 32 kDa) was confirmed by collecting the corresponding fractions and analyzing them by SDS-PAGE, as shown in the gel images in Fig 2. These results revealed that the majority of pea proteins have a molecular weight around 64 kDa, while smaller (32 and 13 kDa) and larger proteins (138, 233, 327, and 458 kDa) are also present at various amounts. Detected proteins with molecular weight around 64 kDa could be a mixture of albumins, legumin and con-vicilin subunits [17]. Proteins with molecular weights of 138, 233, and 327 kDa are probably the vicilin trimer, con-vicilin trimer, and legumin hexamer, respectively [17]. Interestingly, two very early eluting peaks with very high weights were also present (Fig 2B). Because of the presence of BME, a reducing agent, it is unlikely that these proteins are high molecular weight disulfide linked proteins. Instead, these proteins are hypothesized to be glycolated proteins.

### 3.2 2D separation of proteins

**Fig 3:**
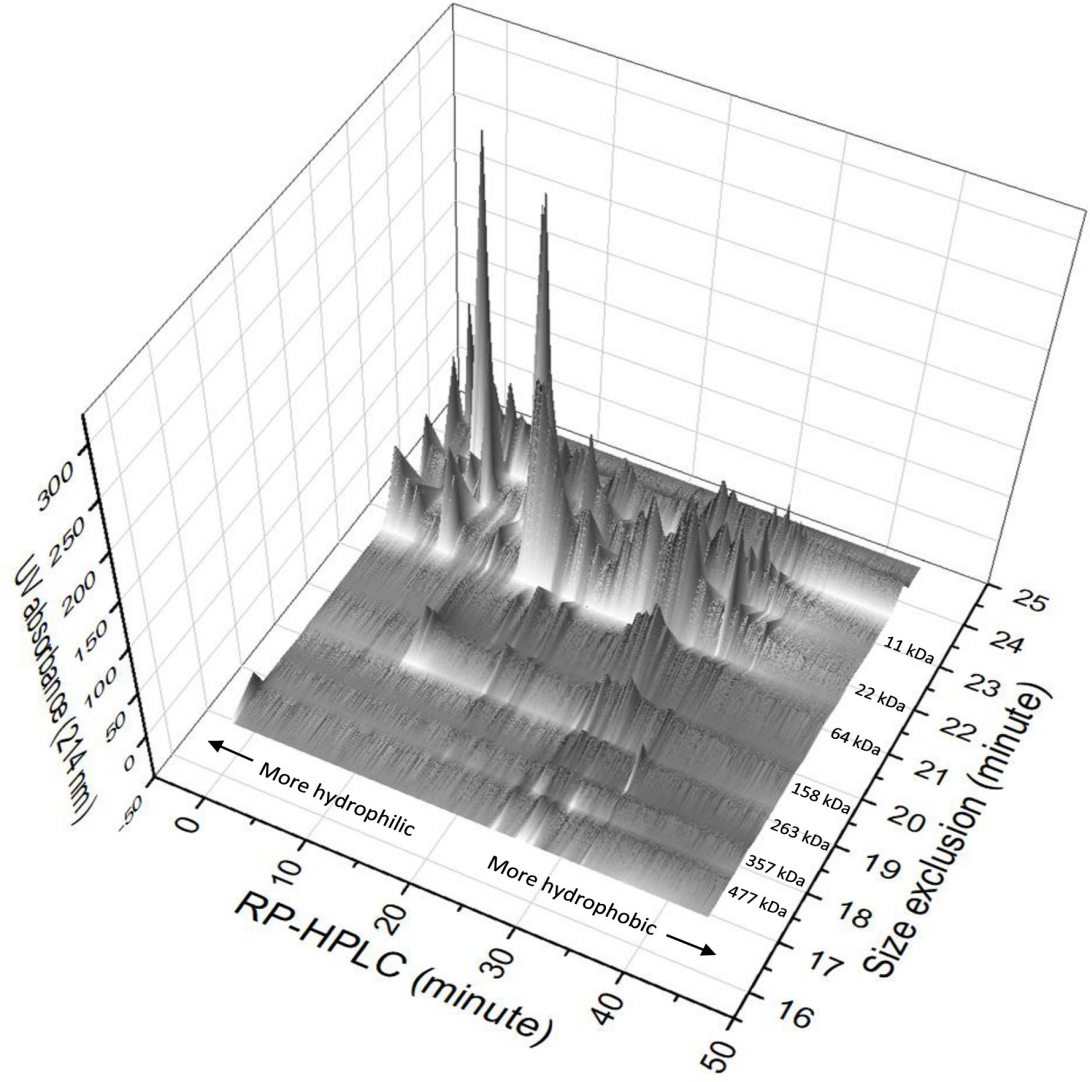
The 3D surface plot of pea proteins separated by SEC (X axis) and RP-HPLC (Y axis).

Fractions of the 7 identified SEC peaks, plus two last peaks representing proteins smaller than 13 kDa, were collected and analyzed by RP-HPLC to further separate those proteins based on their polarity. Combining the RP-HPLC chromatograms of each fraction of SEC, Fig 3 shows a 3D surface plot representation of the resolved proteins. From this, it is evident that each SEC peak contains several proteins with different polarities, which were further separated by RP-HPLC. This means that there are several different proteins in yellow peas with similar molecular weights, which could either be proteins in their native structure, or subunits of larger proteins. Looking at the plot from RP-HPLC angle, some overlapping of peaks could be observed (proteins with different molecular weights are showing similar retention during RP-HPLC). This could be due to the presence of similar amino acid sequences exposed to the outside of some proteins, giving them similar hydrophobicity during RP-HPLC [18]. Regarding the non-polar nature of the stationary phase, early eluting proteins show more hydrophilic characteristics during RP-HPLC [15]. It is noticeable that smaller proteins are more hydrophilic, and proteins with higher molecular weights (above 64 kDa) are more hydrophobic (Fig 3). Generally, the plot shows the majority of yellow pea proteins have molecular weights lower than 64 with more hydrophilic structure, which could be due to the fact that BME successfully cleaved most disulfide bonds resulting in division of larger proteins into their corresponding subunits [6].

### 3.3 RP-HPLC

**Fig 4:**
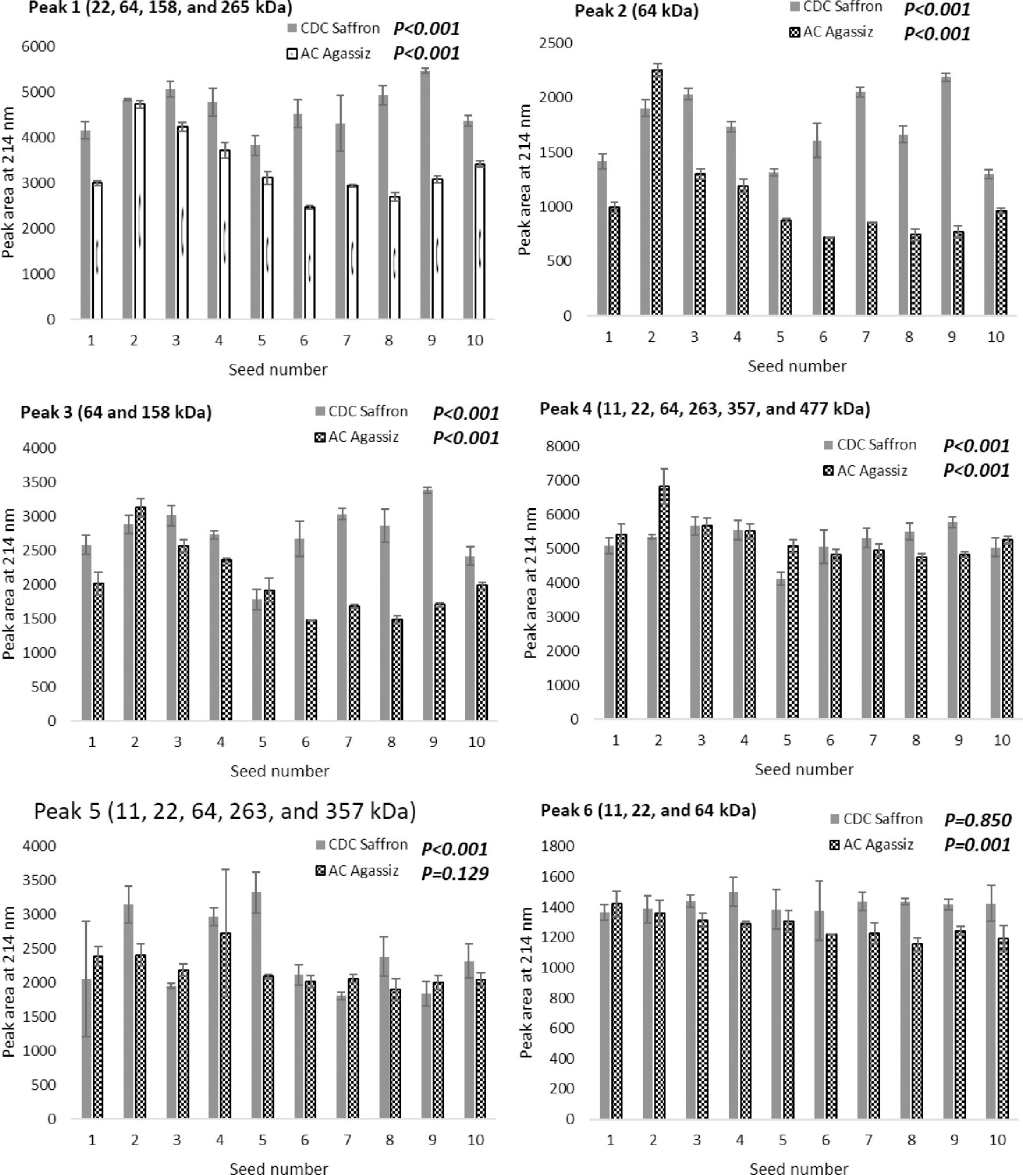
RP-HPLC areas of six selected peaks from analyzing 10 single seeds of both CDC Saffron and AC Agassiz varieties. Results are means of three replicates and error bars represent standard deviation. P represents *P value* for each peak and each variety.

RP-HPLC was first used to estimate protein content of the single seeds. Results showed that protein content was significantly different between two selected varieties (ANOVA; F_1, 39_ = 12.746, *P value = 0*.*001*) as well as among individual seeds (ANOVA; F_9, 39_ = 32.094, *P value<0*.*001*) and variety/seed interaction was also significant (ANOVA; _F9, 39_ = 17.692, *P value<0*.*001*) (Table 1). Through the 2D separation of proteins, the molecular weights of the majority of peaks on RP-HPLC were identified. Multivariate Analysis of Variance (MANOVA) showed the abundance of the six selected RP-HPLC peaks were significantly different between the two varieties (Pillai’s Trace; F_6,34_ = 621.836, *P value<0*.*001*), and among the individual seeds (Pillai’s Trace; F_54,234_ = 4.614, *P value<0*.*001*). The interaction between variety and seed number was also significant (Pillai’s Trace; _F54,234_ = 4.151, *P value<0*.*001*). For AC Agassiz, peaks 1–4 and peak 6 showed significant differences (all with a *P value<0*.*001*), while peak 5 was not significantly different among the 10 selected seeds (*P value = 0*.*129*, Fig 4). For CDC Saffron, peaks 1–5 showed significant differences among the 10 selected seeds (all with a *P value<0*.*001*), while peak 6 was not significantly different among the seeds (*P value = 0*.*850*, Fig 4). It is obvious that variations exist, not only between two varieties, but also among seeds of the same variety. Peak 2 (64 kDa) shows the highest variation both among seeds and varieties (Fig 4). Although no linear correlation was observed with seed weight and size, peaks 1, 2, and 3 appears to have a similar trend from the smallest (seed 1) to the largest (seed 10) seed. While peaks 4, 5, and 6 (more hydrophobic) show lower variations without any obvious correlation. A high variation of proteins among different yellow pea varieties was previously observed in a study by Tzitzikas, Vincken, De Groot, Gruppen, & Visser [7]. For example, the authors reported that among 59 different pea varieties, vicilin content varied from 26.3 to 52.0%. Confirming their study, our results show that this high variability also exists among the individual seeds of the same variety grown at the same location and time. Furthermore, in cowpeas, is has been shown that intra-plant variations in single seed weight and protein content might be associated biased carbon and nitrogen source/sink ratios caused by non-uniform flowering [19], which may help account for variability reported in the present study.

**Table 1:** The weight, average diameter, and total protein content of 10 seeds of each variety. Results are means of three replicates ± standard deviation.

Variety	Seed #	Weight (g)	Average Diameter (mm)	Total protein % ± SD
**AC Agassiz**	**1**	0.1236	5.58	26.4 ± 1.1^b,c,d^
	**2**	0.1387	5.95	35.7 ± 1.7^a^
	**3**	0.149	6.00	29.2 ± 1.0^b^
	**4**	0.1669	6.31	28.4 ± 0.6^b,c^
	**5**	0.1846	6.26	24.3 ± 1.4^d,e,f^
	**6**	0.1822	6.37	21.5 ± 0.2^f^
	**7**	0.2013	6.53	23.6 ± 0.4^d,e,f^
	**8**	0.2172	6.70	21.8 ± 0.8^f^
	**9**	0.2435	7.09	23.1 ± 0.8^e,f^
	**10**	0.3047	7.61	25.7 ± 0.7^c,d,e^
			
	**1**	0.1622	6.10	26.4 ± 1.8^a,b,c^
	**2**	0.1721	6.25	28.8 ± 0.6^a,b^
**CDC Saffron**	**3**	0.1688	6.31	29.5 ± 1.6^a,b^
	**4**	0.1933	6.50	29.3 ± 1.4^a,b^
	**5**	0.2051	6.69	21.7 ± 1.0^d^
	**6**	0.2388	6.90	25.6 ± 2.4^b,c,d^
	**7**	0.2500	7.14	27.4 ± 0.8^a,b,c^
	**8**	0.2418	7.11	27.7 ± 1.5^a,b,c^
	**9**	0.2625	7.19	30.1 ± 0.8^a^
	**10**	0.3070	7.57	24.6 ± 1.3^c,d^

Different letters show significant differences in each variety.

### 3.4 SDS-PAGE

**Fig 5:**
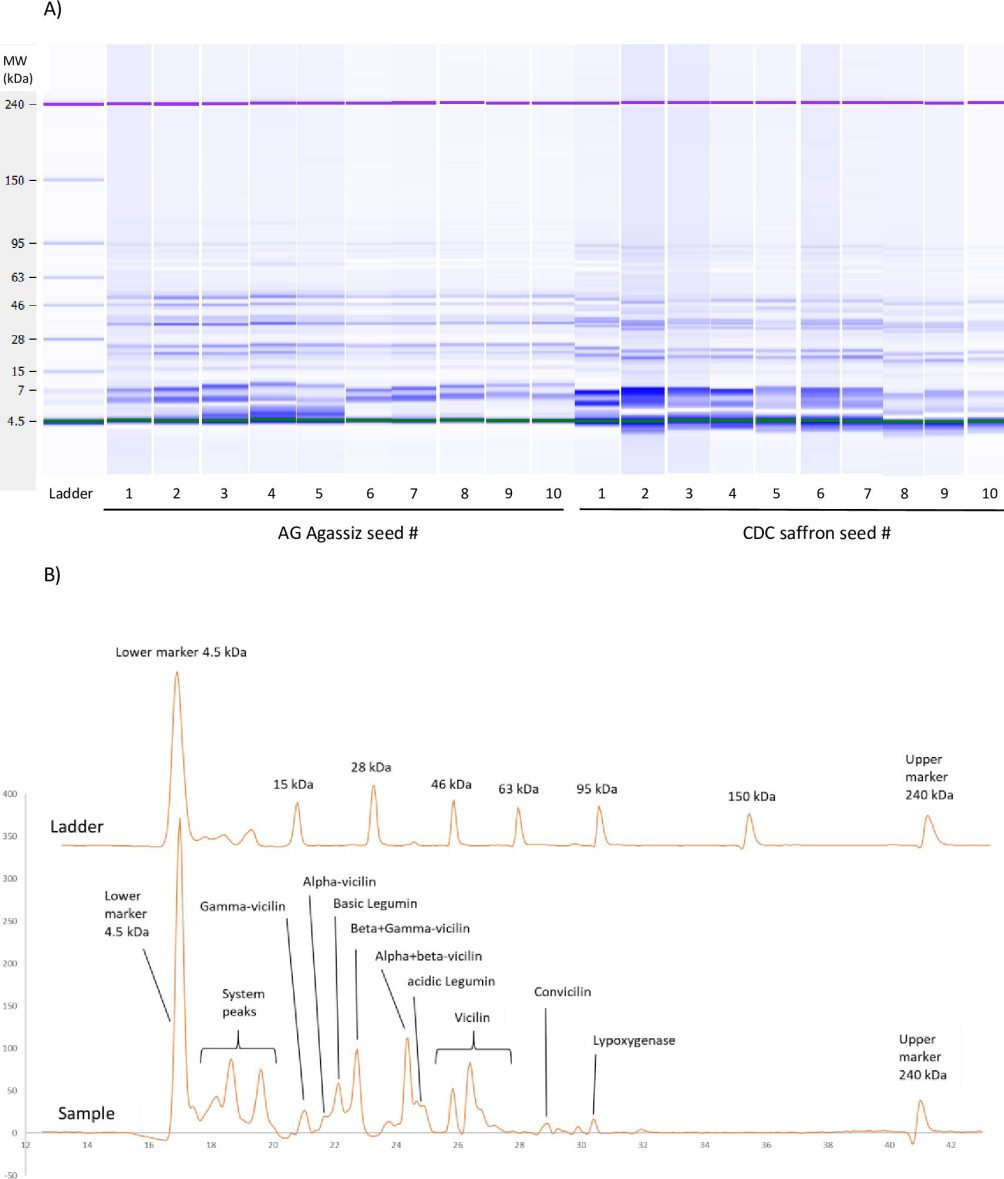
A: The microfluidic SDS-PAGE gel pictures of extracted proteins from 10 single seeds of both CDC Saffron and AC Agassiz varieties. B: A sample electrogram obtained from the microfluidic SDS-PAGE analysis, including the ladder and identification of peaks based on molecular weights.

Fig 5A shows the SDS-PAGE gels of all ten seeds of each variety, where the protein fragments can be observed and identified by their molecular weights. Identifications of proteins (Fig 5B) were made based on the previously reported molecular weights of legumin, vicilin, and con-vicilin subunits [20]. It is apparent that all samples share a similar protein profile, but with varying quantities.

Multivariate Analysis of Variance (MANOVA) showed that protein fractions were significantly different between the two selected varieties (Pillai’s Trace, F_10,10_ = 1181.626, *P value<0*.*001*), and among 10 selected seeds (Pillai’s Trace, F_90,162_ = 4.736, *P value<0*.*001*). The interaction between variety and seed number was also significant (Pillai’s Trace, F_90,162_ = 4.976, *P value<0*.*001*). Gamma-vicilin (GV; *P value = 0*.*001*), alpha-vicilin (AV; *P value<0*.*001*), basic legumin (BL; *P value = 0*.*004*), beta+gamma-vicilin (BGV; *P value<0*.*001*), alpha+beta-vicilin (ABV; *P value<0*.*001*), acidic legumin (AL; *P value<0*.*001*), vicilin (V; *P value<0*.*001*), convicilin (CoV; *P value<0*.*001*), lipoxygenase (LOxy; *P value<0*.*001*), total legumin (*P value<0*.*001*), total vicilin (*P value<0*.*001*), and total vicilin/total legumin ratio (V/L; *P value<0*.*001*) were significantly different between the two varieties.

For AC Agassiz alone, variations among seeds were significant (Pillai’s Trace, F_90,81_ = 1.420, *P value = 0*.*050*). GV (*P value = 0*.*002*), AV (*P value = 0*.*002*), BGV (*P value<0*.*001*), AL (*P value = 0*.*031*), V (P value = 0.004), LOxy (*P value = 0*.*003*), total legumin (*P value = 0*.*014*), total vicilin (*P value = 0*.*014*), V/L ratio (*P value = 0*.*005*) were significantly different (Table 2). While BL, ABV, and copnvicilin were not significantly different among the selected seeds. For CDC Saffron alone, variations among seeds were significant (Pillai’s Trace, F_81,81_ = 1.925, P value = 0.002). AV (*P value = 0*.*006*), BL (*P value<0*.*001*), BGV (*P value = 0*.*009*), vicilin (*P value<0*.*001*), CoV (*P value = 0*.*014*), total legumin (*P value = 0*.*002*), total vicilin (*P value = 0*.*027*) and V/L (*P value = 0*.*006*) were significantly different (Table 2). While GV, AL, and LOxy were not significantly different among the selected seeds.

**Table 2:** Percentage of different protein fractions/subunits for ten individual seeds for two varieties. Mean, standard deviation, range, CV and P value were provided.

Variety		Protein fraction/subunit^2^											
		GV	AV	BL	BGV	ABV	AL	V	CoV	LOxy	Total Legumin	Total Vicilin	V/L
AC Agassiz	Mean^1^ (percentage)	4.92	3.88	12.79	17.99	15.76	9.32	25.95	1.66	2.11	22.11	68.49	3.15
	Standard deviation	1.00	0.85	2.06	3.84	1.35	0.76	3.56	0.43	0.93	2.59	2.18	0.49
	Range	3.1	3.20	7.30	13.90	4.90	2.70	15.10	1.40	2.80	10.00	8.50	1.86
	CV (%)	20.3	22.0	16.1	21.4	8.5	8.1	13.7	26.0	44.0	11.7	3.2	15.6
	P value	**0.002**	**0.002**	**0.057**	**<0.001**	**0.439**	**0.031**	**0.004**	**0.080**	**0.003**	**0.014**	**0.014**	**0.005**
CDC Saffron	Mean^1^ (percentage)	3.09	24.49	13.90	3.37	11.35	14.56	13.30	2.05	2.83	28.46	55.60	1.96
	Standard deviation	1.22	2.34	1.33	0.69	1.96	1.45	2.53	0.32	0.88	2.02	2.44	0.17
	Range	5.10	8.90	4.50	2.80	7.10	5.10	8.30	1.30	3.00	6.90	10.60	0.58
	CV (%)	39.3	9.5	9.60	20.6	17.2	9.9	19.0	15.7	31.1	7.1	4.4	8.4
	P value	**0.648**	**0.006**	**<0.001**	**0.009**	**0.001**	**0.059**	**<0.001**	**0.014**	**0.067**	**0.002**	**0.027**	**0.006**
P value between varieties^3^		**<0.001**	**<0.001**	**0.004**	**<0.001**	**<0.001**	**<0.001**	**<0.001**	**<0.001**	**0.002**	**<0.001**	**<0.001**	**<0.001**

^1^ Mean, standard deviation, range, coefficient of variation (CV) and P value were obtained from the ten selected individual seeds for each variety.

^2^ GV: gamma-vicilin; AV: alpha-vicilin; BL: basic legumin; BGV: beta+gamma-vicilin; ABV: alpha+beta-vicilin; AL: acidic legumin; V: vicilin; CoV: convicilin; LOxy: lipoxygenase; total legumin: BL+AL; total vicilin: GV+AV+BGV+ABV+V; V/L: total vicilin/total legumin.

^3^ These P values compare differences between the two selected varieties.

The SDS-PAGE results showed high variations between the quantities of each protein/protein fragment and different patterns between the two selected varieties. For AC Agassiz, the beta+gamma-vicilin had the highest percentage of vicilin fragments, which varied between 11.1 to 24.1% (mean17.99%, CV = 21.4%) among the seeds, and alpha+beta-vicilin as the second abundant vicilin fragment showed relatively similar percentage among the seeds (14.5–17.8%). For AC Agassiz, lipoxygenase showed the highest variation (CV = 44.0%) which was followed by convicilin (CV = 26.0%). V/L ratio showed 15.6% variation among the selected seeds. The variability of the proteins is of tremendous importance in understanding and determining application, as well as figuring out underlying reasons for product variability in food processing situations. The 7S/11S (vicilin/legumin) protein ratio has been reported to be between 1.2 and 8.0 in peas [7]. This ratio for soy glycinin and β-conglycinin are far less variable and is known to play an important role in protein functionality such as gelling characteristics, where higher portion of the 11s subunit in soy proteins are known to produce firmer gels. Conversely, lower ratios will produce a smoother less firm gel [12]. Total legumin for the AC Agassiz was 17.3–24.8% and total vicilin was 65.3–72.2%. The total values of each fraction also showed high variations among the seeds. Vicilin to legumin ratio was between 2.72–4.19 with a mean of 3.15 among the seeds investigated in this study, which is in range of the values reported by Lam, Warkentin, Tyler, & Nickerson [21].

For CDC Saffron, alpha-vicilin showed the highest percentage of vicilin fragments, which varied between 21.8 to 28.7% (mean = 24.49%, CV = 9.5%), and alpha+beta-vicilin as the second abundant vicilin fragment was in range of 6.9–13.4% (mean = 11.35%, CV = 17.2%). Total legumin (25.6–31.65%) and total vicilin (53.3–60.55%) also showed high variations among the seeds. Vicilin to legumin ratio was between 1.70–2.22 with a mean of 1.96 among the CDC Saffron seeds. While the results from Lam et al. [21] were reported as legumin to vicilin, the results showed similar trends as those reported here. When values from this study are converted to vicilin to legumin ratio, CDC varieties (Dakota, Golden, Striker, and Tetris) and Cooper ranged from ~1.23–1.66 and the Agassiz was ~2.63.

It is obvious that vicilin fragments differ in quantity between the two varieties, which led to higher beta+gamma-vicilin fragment in AC Agassiz and higher alpha-vicilin fragment in CDC Saffron. To evaluate the impact of these protein fragments on functionality of the protein isolates, vicilin to legumin (V/L) ratio can be used. V/L ratio was shown to be important in defining both the nutritional value (i.e. lysine availability), and the functionality (i.e. solubility, interfacial, textural, sensory, foaming and emulsifying properties) of pea proteins [22–25]. Vicilin has shown superior interfacial properties compared to legumin [25], and it formed firmer gels and more stable foams and emulsions [23, 24]. The purpose of this study was only to determine variability between seeds and if this variability correlated to physical parameters that could be used for sorting. To investigate varietal differences in vicilin to legumin ratios that could be used to determine potential food applications, a much larger varietal screening is needed like the recent study completed by Choi, Taghvaei, Smith, & Ganjyal [26]. In this study 47 unique cultivars were investigated and only SW Midas was found to have a higher vicilin to legumin ratio than AC Agassiz, which were 2.93 and 2.91 respectively. Therefore, with a high vicilin to legumin ratio in the Agassiz variety being reported in the present study, by Choi et al. [26], and by Lam et al. [21], this variety is likely a better choice for protein isolation for food applications.

For AC Agassiz, significant correlation was observed between seed weight and beta+gamma-vicilin (r = 0.691, *P value = 0*.*027*), vicilin (r = -0.774, *P value* = 0.009) and vicilin fragments (r = 0.741, *P value* = 0.014). On the contrary, no correlation was found between weight or size of the seeds and V/L ratio. For CDC Saffron, none of the protein fragments showed any significant correlation with weight or size of the seeds. One limitation to this study was the limited number of batches from where the samples were drawn. While this was done to limit sources of variability (GxE influences), in future studies, increasing the number of varieties, growing locations, and the number of seeds for multiple seasons may be a route to determine inherent variability within a cultivar. Therefore, future work needs to be completed to determine environmental impacts on seed-to-seed protein variability of field peas. By increasing the number of replicates in future works one might be able to determine whether sorting technologies could be applied to manipulate protein content and profile of raw materials for protein isolation purposes.

## 4. Conclusion

A new approach successfully was applied to analyze proteins of yellow pea combining three analytical methods of SEC, RP-HPLC, and microfluidic SDS-PAGE to achieve the highest separation resolution. A high variability of protein content and quantities of protein fragments/subunits was observed not only between two varieties, but also among seeds of the same variety. The existing variations between the two selected varieties of peas could be mainly due to genotype of the varieties, while difference among seed could be mainly related to environmental conditions and maturity of the seeds. While the amount of some proteins in AC Agassiz variety correlated with seeds’ size and weight, such correlations was not observed in CDC Saffron variety.

## Supporting information

S1 Data (XLSX)

S2 Data (XLSX)

S3 Data (XLSX)

S4 Data (XLSX)

S5 Data (CSV)

S6 Data (XLSX)

S7 Data (XLSX)

S8 Data (XLSX)
